# Case of Ununited Fracture of the Radius of about Twelve Weeks’ Standing, Cured by Sub-Cutaneous Perforation of the Bone

**Published:** 1859-05

**Authors:** A. L. McArthur

**Affiliations:** Joliet, Ill.


### ARTICLE IV.

### CASE OF UNUNITED FRACTURE OF THE RADIUS OF ABOUT TWELVE WEEKS' STANDING CURED BY SUB-CUTANEOUS PERFORATION OF THE BONE.

By A. L. McARTHUR, M.D., of Joliet, Ill.

Mr. H—, a laborer, aged thirty-five years, called at my office, October, 1856, to consult me in regard to an ununited fracture of the radius. The history he gave of his case is as follows:

Four months previously, while assisting in raising a building, he fell some fifteen feet, upon his left side and shoulder, fracturing the radius, and bruising the arm and shoulder severely.

The fracture was reduced, and treated by a physician in the neighborhood, in the usual manner, and with apparent success.

After four or five weeks, however, it was ascertained that the bones had not united. The doctor then put the arm into a starch bandage. Five weeks after, they were found, by examination, to be still ununited. A consultation was had, and it was decided to irritate the bones by rubbing them together, in order to get up an adhesive inflammation. This was done, and the arm put in a starch bandage. After the lapse of three or four weeks, the bones were found ununited.

The appearance of the limb when I first saw it, was soft, flabby, and atrophied, with a slightly oblique fracture of the radius, a little above the union of the middle and lower third. The usual modes of treating such fractures having been thoroughly tried, and failed, I resorted to the treatment by perforation, practiced and recommended by Professor Brainard. Procuring an instrument similar to an awl, save the end, which was flattened, I perforated the soft parts directly over the fracture, passing the instrument between the ends of the bones, nearly to the opposite side, the flattened surfaces of the instrument being at a right angle with the surfaces of fracture. The instrument was then withdrawn from the bones, which being turned to the right, and the soft parts to the left, the instrument was thrust through the fracture at a new point. Withdrawing the instrument from the fracture, the operation was reversed, by rotating the bones to the left, and the soft parts to the right, and the instrument passed nearly through again. The limb was then loosely dressed in the ordinary way, with splints and roller. On the following day, contrary to expectation, the limb appeared as before; no apparent inflammation or swelling; slight tenderness at the point of perforation. I dressed the forearm with a starch bandage, and the man returned to his home in the country. Four or five weeks after, he returned, the bandages were removed, and the bones were found to have united. He carried the arm in a sling for a few weeks, and, greatly to his surprise and gratification, he had a sound and useful limb.

